# Chemical Exploration of a Highly Selective Scaffold with Activity against Intracellular *Mycobacterium tuberculosis*

**DOI:** 10.1128/spectrum.01161-22

**Published:** 2022-05-25

**Authors:** Samuel Njikan, Sara Ahmed, Alyssa Manning, Divya Awasthi, Yulia Ovechkina, Sultan Chowdhury, Arielle Butts, Tanya Parish

**Affiliations:** a Infectious Disease Research Institute, Seattle, Washington, USA; b Center for Global Infectious Disease Research Seattle, Seattle Children’s Research Institute, Seattle, Washington, USA; Johns Hopkins University School of Medicine

**Keywords:** high-content analysis, *Mycobacterium tuberculosis*, phenotypic screening, antibacterial drug discovery

## Abstract

We previously identified a phenylthiourea series with activity against intracellular Mycobacterium tuberculosis using a high-throughput, high-content assay. We conducted a catalog structure-activity relationship study with a collection of 35 analogs. We identified several thiourea derivatives with excellent potency against intracellular bacteria and good selectivity over eukaryotic cells. Compounds had much lower activity against extracellular bacteria, which was not increased by using cholesterol as the sole carbon source. Compounds were equally active against strains with mutations in QcrB or MmpL3, thereby excluding common, promiscuous targets as the mode of action. The phenylthiourea series represents a good starting point for further exploration to develop novel antitubercular agents.

**IMPORTANCE** Mycobacterium tuberculosis is responsible for the highest number of deaths from a bacterial pathogen, with >1.5 million in 2020. M. tuberculosis is a sophisticated pathogen that can replicate inside immune cells. There is an urgent need for new drugs to combat M. tuberculosis and to shorten therapy from 6 to 24 months. We have identified a series of molecules that inhibit the growth of M. tuberculosis inside macrophages; we tested a number of derivatives to link structural features to biological activity. The compounds are likely to have novel mechanism of action and so could be developed as new agents for drug-resistant tuberculosis.

## INTRODUCTION

Despite decades of extensive research efforts toward diagnosis, vaccination, and treatment, tuberculosis remains one of the leading infectious causes of death globally. The World Health Organization estimates that nearly one in four people are latently infected with Mycobacterium tuberculosis, and there were 1.5 million deaths in 2020 (1). Infection with M. tuberculosis generally occurs through the inhalation of aerosolized bacteria released by those with active tuberculosis. Bacteria are phagocytosed in the lower respiratory tract by alveolar macrophages (2). In most cases, this results in a latent infection and the individual remains asymptomatic and noncontagious. However, in some cases, active symptomatic disease develops, and reactivation can occur as a result of immune suppression or increased immune stress (3).

While there are therapeutic interventions available for the treatment of disease, they leave much to be desired. Treatment for drug-susceptible M. tuberculosis requires a multidrug regimen taken over a period of 6 months. Side effects of the individual components, drug-drug interactions, and the length of treatment dramatically impact compliance, thereby reducing cure rates and contributing to the emergence of drug resistance (4). Treatment of drug-resistant strains requires more complex regimens over a prolonged period, further reducing the likelihood of a successful outcome (5). The need for a complex treatment regimen for tuberculosis is rooted in the complex and heterogenous nature of the disease. During infection, M. tuberculosis exists in the host in several microenvironments and metabolic states, which need to be targeted during treatment (6).

While target-based drug discovery efforts are experiencing a resurgence in the field of antibacterial drug discovery, and in tuberculosis research in particular, the majority of successful agents are still identified through more traditional whole-cell screening approaches (7, 8). An understanding of the properties that contribute to cellular penetration and uptake is still lagging in the field of bacteriology, compared to our extensive understanding of the features when it comes to mammalian cell penetration (9). Antimycobacterials must also penetrate the waxy mycomembrane. Identifying novel scaffolds requires venturing into previously unexplored chemical spaces or the development of unique screening strategies with improved sensitivity and/or different specificity.

A key feature of M. tuberculosis infection that contributes significantly to both pathogenesis and treatment difficulty is its ability to survive and proliferate inside macrophages (10). In the context of treatment, this means that active compounds must access this intracellular compartment to exert their antibacterial activity. This requirement is rarely considered during the compound screening phase if compounds are examined against axenically grown bacteria. To address this limitation, we developed a high-content, high-throughput screening assay to determine activity of compounds against M. tuberculosis growing within mammalian macrophages (11). Not only does this approach facilitate the prioritization of compounds with intracellular activity, but it also enables us to filter based on macrophage survival and remove cytotoxic compounds from follow-up studies. Additionally, this expands the realm of potential targets to include those that are conditionally essential under the physiologically relevant conditions experienced during macrophage infection which are overlooked when screening against bacteria grown in rich medium (12–14).

We previously screened a diversity collection of 10,000 compounds and identified the phenylthiourea (PTU) series as active against intracellular bacteria (15). Ureas and thioureas are useful in a variety of diseases (16–19), since they have good pharmacokinetic properties, including solubility and permeability. Several studies have demonstrated the antimycobacterial properties of thiourea and urea (20). In their simplest form, urea, thiourea, and guanidine are structurally similar, although the difference in heteroatoms imparts different physicochemical properties, including basicity; urea and thiourea are more neutral functional groups, whereas guanidine is a strong base. Both moieties have hydrogen-bond donors (from the nitrogens) and hydrogen-bond acceptors via the thio/carbonyl, which facilitates water solubility. Phenyl thioureas generally have good physicochemical profiles with low lipophilicity and low molecular weights, as well as facile synthetic routes. Based on these properties in this study, we have further investigated this series to determine its potential for development as a novel antitubercular agent.

## RESULTS

### Series identification and expansion.

**FIG 1:**
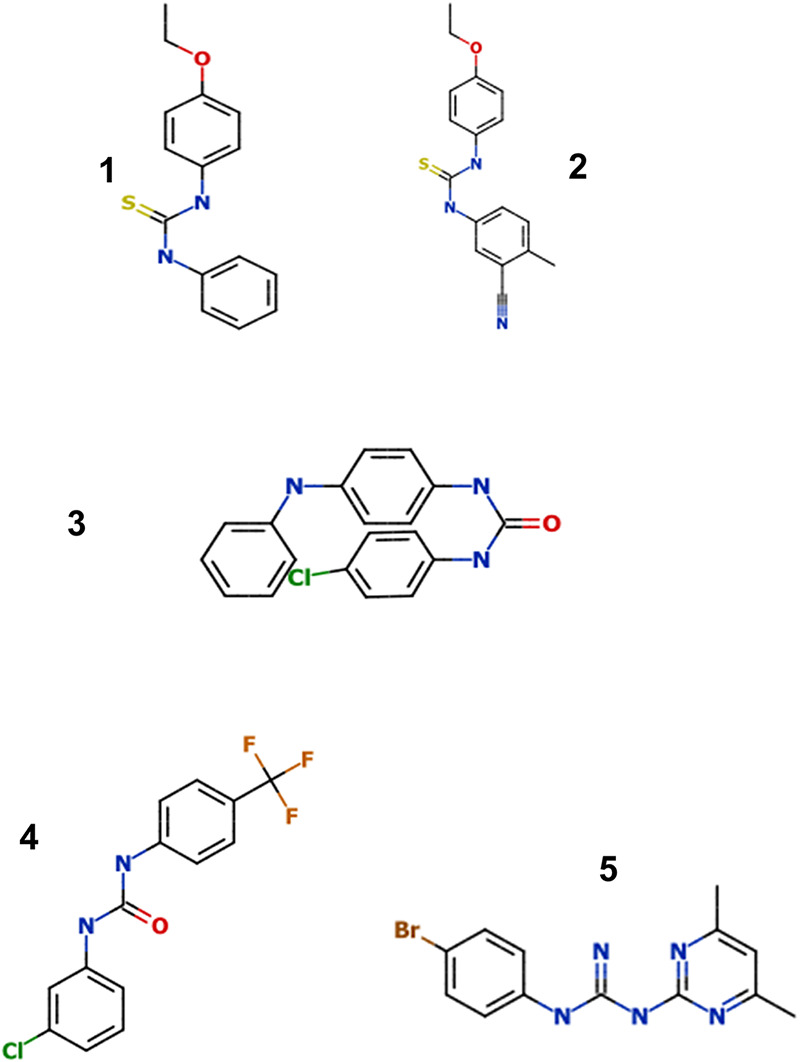
Structure of compounds identified in the screen.

We previously demonstrated that PTU analogs have some potential as antitubercular agent, since our previous work demonstrated that 4/5 analogs in the screening collection had activity (Fig. 1 and Table 1) (15). Three of these compounds (1, 2, and 5) had excellent potency and selectivity for M. tuberculosis over eukaryotic cells (Table 1) (15). The thiourea analogs demonstrated activity against extracellular bacteria but were 5- to 10-fold less active (15). Compounds 1 and 2 were thioureas with excellent intracellular activity (50% inhibitory concentration [IC_50_] of 0.4 and 0.3 μM, respectively) (15). Since the series targeted both intracellular and extracellular bacteria, we considered this to be a good starting point for exploration.

**TABLE 1:** Phenylthiourea hits identified from the primary screen

Compound	Macrophage IC_50_ (μM)^*a*^	Intracellular TB IC_50_ (μM)^*a*^	Extracellular TB IC_50_ (μM)^*b*^	HepG2 IC_50_ (μM)^*b*^
1	>100	0.40	>20	>100
2	47	0.30	9.5	64
3	68	NC	>20	>100
4	1.8	0.70	15	5.6
5	18	1.3	8.2	22

a Activity against macrophages (RAW 264.7) and intracellular M. tuberculosis was determined using the high-content assay.

b Activity against extracellular M. tuberculosis or HepG2 cells was determined in standard culture medium. IC_50_, concentration required to inhibit by 50%. Data are the average of at least two independent experiments. NC, not calculated due to cytotoxicity. Taken from reference 15.

### Characterization and structure-activity relationships.

**FIG 2:**
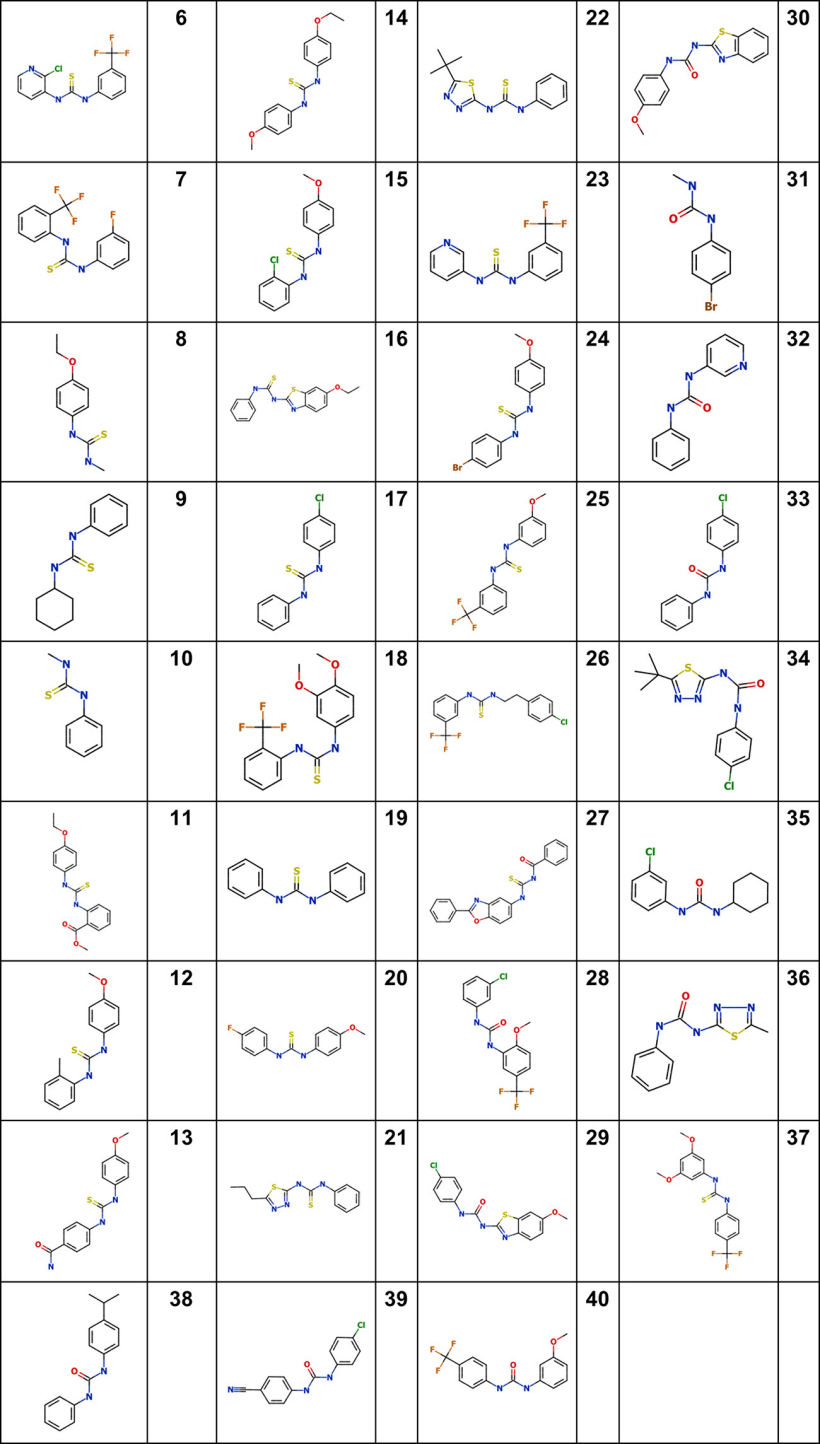
Structure of novel analogs.

We initiated a structure-activity relationship study using commercially available analogs. We identified 35 additional analogs which represented molecules containing either a thiourea core, as present in the initial hits, or a urea core. Modifications on both sides of the central core were explored, including decoration of the phenyl rings, substitutions with other cyclic structures (aromatic and nonaromatic), and removal of one side (Fig. 2 and Table 2).

**TABLE 2:** Biological activity of urea and thiourea analogs

Compound	Macrophage IC_50_ (μM)^*a*^	Intracellular TB IC_50_ (μM)^*a*^	Extracellular TB IC_50_ (μM)^*b*^	HepG2 IC_50_ (μM)^*b*^
6	19	NC	>200	>100
7	53	>100	>200	86
8	>100	10	80	>100
9	>100	>100	130	>100
10	>100	>100	>200	>100
11	46	NC	>200	>100
12	>100	19	>200	>100
13	>100	>100	>200	>100
14	>100	0.17	7.2	>100
15	>100	14	>200	>100
16	28	0.75	26	73
17	42	16	104	91
18	>100	>100	>200	>100
19	>100	15	64	>100
20	>100	9.2	91	>100
21	ND	0.4	14.5	>100
22	>100	0.51	11	>99
23	30	NC	125	62
24	72	2.1	16	>100
25	17	9.6	63	24
26	9.3	NC	30	23
27	80	0.35	>200	>100
28	3	NC	25	12
29	56	NC	>200	>100
30	52	NC	>200	54
31	>100	64	>200	>100
32	>100	>100	>200	>100
33	>100	>100	>200	>100
34	>100	84	>200	>100
35	>100	4.5	28	>100
36	>100	>100	>200	>100
37	29	NC	>200	49
38	85	>100	>200	55
39	3.0	NC	11	6.5
40	5.2	NC	31	11

a Activity against macrophages (RAW 264.7) and intracellular M. tuberculosis was determined using the high-content assay.

b Activity against extracellular M. tuberculosis or HepG2 cells was determined in standard culture medium. IC_50_, concentration required to inhibit by 50%. Data are the average of at least two independent experiments. NC, not calculated due to cytotoxicity.

**FIG 3:**
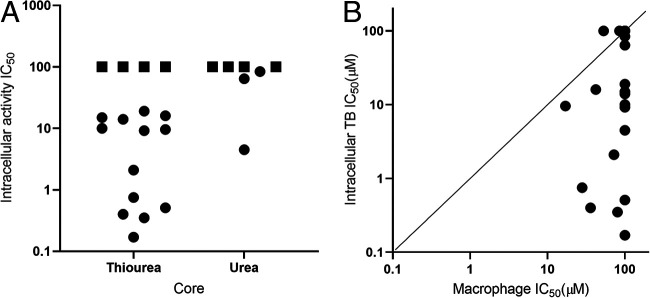
Antitubercular activity of ureas and thioureas against intracellular M. tuberculosis. (A) Antitubercular activity by core. Squares indicate where no activity was seen, i.e., IC_50_ > 100 μM. (B) Activity against M. tuberculosis and murine macrophages. Activity against macrophages (RAW 264.7) and intracellular M. tuberculosis was determined using the high-content assay. IC_50_, concentration required to inhibit by 50%. Data are the average of at least two independent experiments.

We tested all 35 analogs as a 10-point dose response curve using the high-content infection assay (Table 2 and Fig. 3). We determined the IC_50_ (defined as the concentration effecting 50% growth inhibition) against intracellular M. tuberculosis and the infected macrophages in the same samples. Compounds were also tested in 10-point dose response for activity against axenically grown M. tuberculosis and cytotoxicity against the human HepG2 hepatic cell line. Compounds had a good dynamic range of activity from inactive (>100 μM for 9 molecules) to submicromolar (4 molecules) (Fig. 3A). The most potent molecule was 14 with an IC_50_ of 0.17 μM against intracellular M. tuberculosis. Notably, the four most active molecules were all thioureas (Fig. 3A). The activity of 10 molecules, representing both urea and thiourea cores, could not be determined due to cytotoxicity.

Analogs 8, 12, 15, 17, and 19 containing a thiourea pharmacophore exhibited good potency (IC_50_ < 20 μM), and five thiourea compounds (14, 16, 21, 22, and 27) had excellent potency (IC_50_ < 1.0 μM) (Table 2). The common feature in these asymmetrical thiourea analogs is the presence of an electron-rich phenyl ring attached to either side of the thiourea functionality. Interestingly, while several of the analogs were also active against extracellular bacteria, the potency was greatly reduced; for example, compound 14 was the most active, with an IC_50_ of 7.2 μM against extracellular bacteria and an IC_50_ of 0.17 μM against intracellular bacteria (>40-fold difference).

The thiourea containing one phenyl ring with an ethoxy group at the C-4 position of the phenyl ring and an alkyl group (compound 8) was active (IC_50_ = 10 μM), but losing the ethoxy group on the phenyl ring resulted in loss of activity (compound 10, IC_50_ > 100 μM). A similar trend was observed with compound 9, where the absence of the ethoxy moiety on the phenyl ring and a cyclohexyl group present on the thiourea core led to loss of intracellular activity. Ureas containing a substituted phenyl ring and an alkyl chain retained activity (compound 31, IC_50_ = 64 μM and compound 35, IC_50_ = 4.5 μM). Compound 35 had good biological activity, with some extracellular activity (IC_50_ = 28 μM) and no cytotoxicity.

Compounds with varied combinations of electron-withdrawing groups and electron-donating groups at either side of the thiourea moiety showed very different biological activity. Compound 15 with a methoxy group attached to one phenyl ring and a chlorine atom on the other phenyl ring and compound 17 (IC_50_ = 16 μM) which lacked the methoxy were equally active (IC_50_ of 14 and 16 μM, respectively). Compound 13 with amide and methoxy groups was inactive, as was compound 18 with a trifluoromethyl group and two electron-rich methoxy groups. However, similar analogs with different combinations were active; analogs 20 and 25 with a strong electron-withdrawing group (fluorine or trifluoromethyl) on one phenyl ring and an electron-rich methoxy group on the other phenyl ring had activity (IC_50_ = 9.2 μM and IC_50_ = 9.6 μM, respectively). It is worth mentioning in this perspective that the attachment of the trifluoromethyl group was different in each compound. However, replacement of the strong electron-withdrawing fluorine atom with a weaker electron-withdrawing group (bromine; compound 24) improved activity substantially (5-fold increase in potency, IC_50_ = 2.1 μM). A phenyl ring with electron-withdrawing groups on either end of the thiourea functionality resulted in cytotoxicity to macrophages (compounds 6 and 23). Taken together, these data suggest that compounds with an electron-rich group attached at the 4 position of the phenyl are more potent than substituents attached at either the 3 or the 2 position.

Another interesting feature in this series is seen with compounds 21, 22, and 34; these are all asymmetrical analogs containing a 1,3,4-thiadiazole with a lipophilic chain attached at the C-5 position. Compounds 21 and 22 with the thiourea functionality are potent (IC_50_ = 0.40 μM and IC_50_ = 0.51 μM, respectively), whereas compound 34 with a urea group lost activity. The presence of an unsubstituted phenyl ring and a heteroaromatic 1,3,4-thiadiazole ring at one end of the thiourea functional group provides a future opportunity to explore substitutions in different positions as well as to expand to other 5-member heteroaromatic ring systems in order to improve drug-like properties of the molecule. A direct comparison between compound 22 (IC_50_ = 0.51 μM) and compound 34 (IC_50_ = 84 μM) reveals the requirement for the thiourea moiety.

Compound 27 (IC_50_ = 0.35 μM) has a unique acyl thiourea functional group. The asymmetrical acyl thiourea provides a handle to explore both sides of the molecule by addition of different functional moieties to improve lipophilicity and molecular weight. This opens possibilities to prepare derivatives of compound 27 in order to explore different 5- and 6-member rings to improve physicochemical properties.

### Cytotoxicity against HepG2 correlates with cytotoxicity against RAW 264.7 cells.

**FIG 4:**
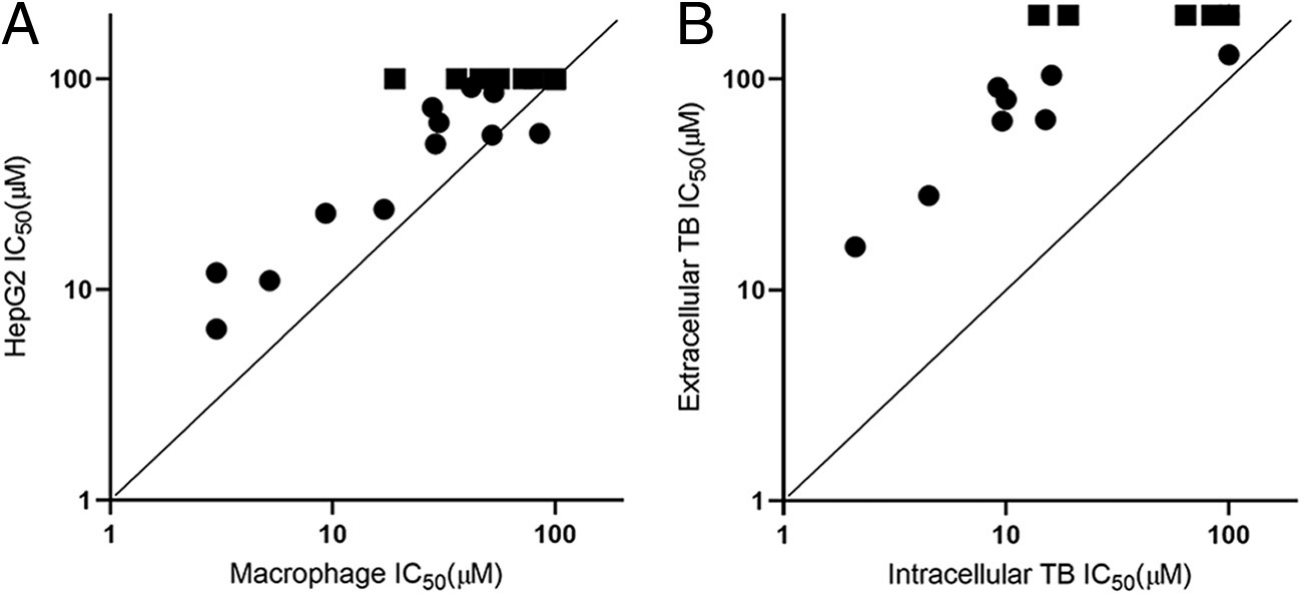
Biological profile of PTU series. (A) Comparison of cytotoxicity against RAW267.4 and HepG2 cells. (B) Comparison of activity against intracellular and extracellular M. tuberculosis. Activity against macrophages (RAW 264.7) and intracellular M. tuberculosis was determined using the high-content assay. Activity against extracellular M. tuberculosis or HepG2 cells was determined in standard culture medium. IC_50_, concentration required to inhibit by 50%. Data are the average of at least two independent experiments. Squares indicate where no activity was seen, i.e., IC_50_ > 100 μM (>200 μM for the extracellular bacterial assay).

There was a strong correlation between cytotoxicity against HepG2 cells and infected macrophages (Fig. 4A). Several compounds showed a combination of potency against intracellular M. tuberculosis and good selectivity (Table 2 and Fig. 3B). For example, compound 14 has submicromolar activity (IC_50_ = 0.17 μM) with a lack of cytotoxicity against either cell line (IC_50_ > 100 μM) giving a selectivity index (SI) of 588. Similarly, compounds 21, 22, and 27 all had an SI of >200. Seventeen compounds showed no activity against either eukaryotic cell line (Table 2). In addition, activity against M. tuberculosis was not correlated with cytotoxicity, demonstrating that the series does not have a cytotoxicity issue.

### PTU compounds are more active against intracellular bacteria.

We tested all analogs for activity against axenically cultured M. tuberculosis (Table 2 and Fig. 4B). Several of the analogs with intracellular activity were also active against extracellular bacteria but with much lower potency, ranging from a 4.3-fold difference (19 with intracellular IC_50_ of 15 μM and extracellular IC_50_ of 64 μM) to a >20-fold difference (22 with intracellular IC_50_ of 0.51 μM and extracellular IC_50_ of 11 μM). Compound 27 had the largest differential activity of >500-fold with intracellular IC_50_ of 0.35 μM and no extracellular activity (IC_50_ > 200 μM).

### PTUs exhibit minimal activity against extracellular bacteria.

**FIG 5:**
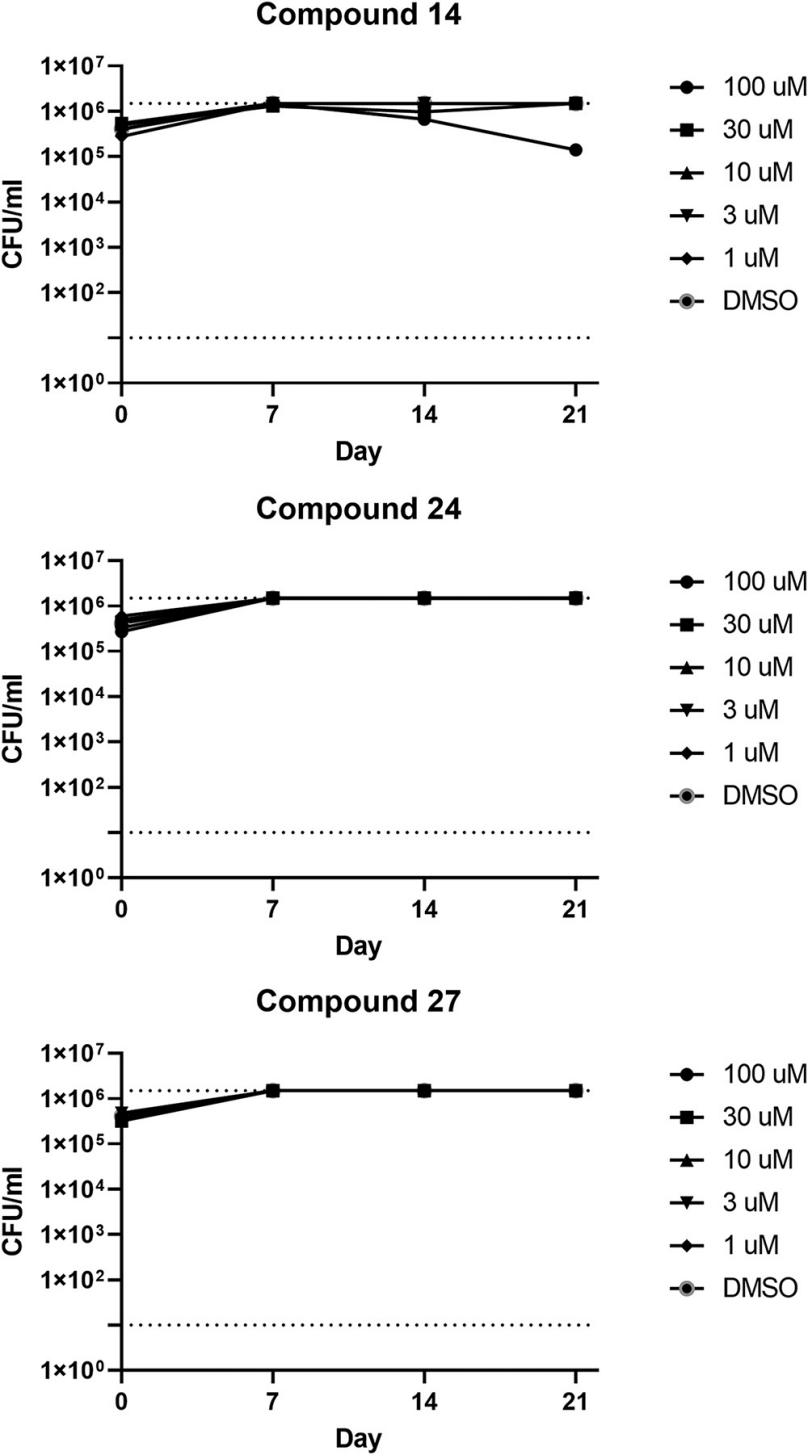
Kill kinetics of representative compounds against replicating M. tuberculosis. M. tuberculosis was exposed to compounds under replicating conditions (aerobic culture) in 7H9-Tw-OADC. Viability was monitored by determining CFU.

To further assess the activity of this series, three analogs with a broad range of potencies were selected for kill kinetics under standard replicating conditions (Fig. 5). Compounds were tested up to 100 μM with minimal impact on culture viability over 21 days. Even at the highest concentrations, there was no inhibition of growth in the first 7 days, consistent with the results seen in the 5-day assay. One compound (14) resulted in a small decrease in viable bacteria at day 21 at the highest concentration. Thus, we confirmed that compounds have minimal activity against axenically cultured bacteria.

### Target and mechanism of action.

**FIG 6:**
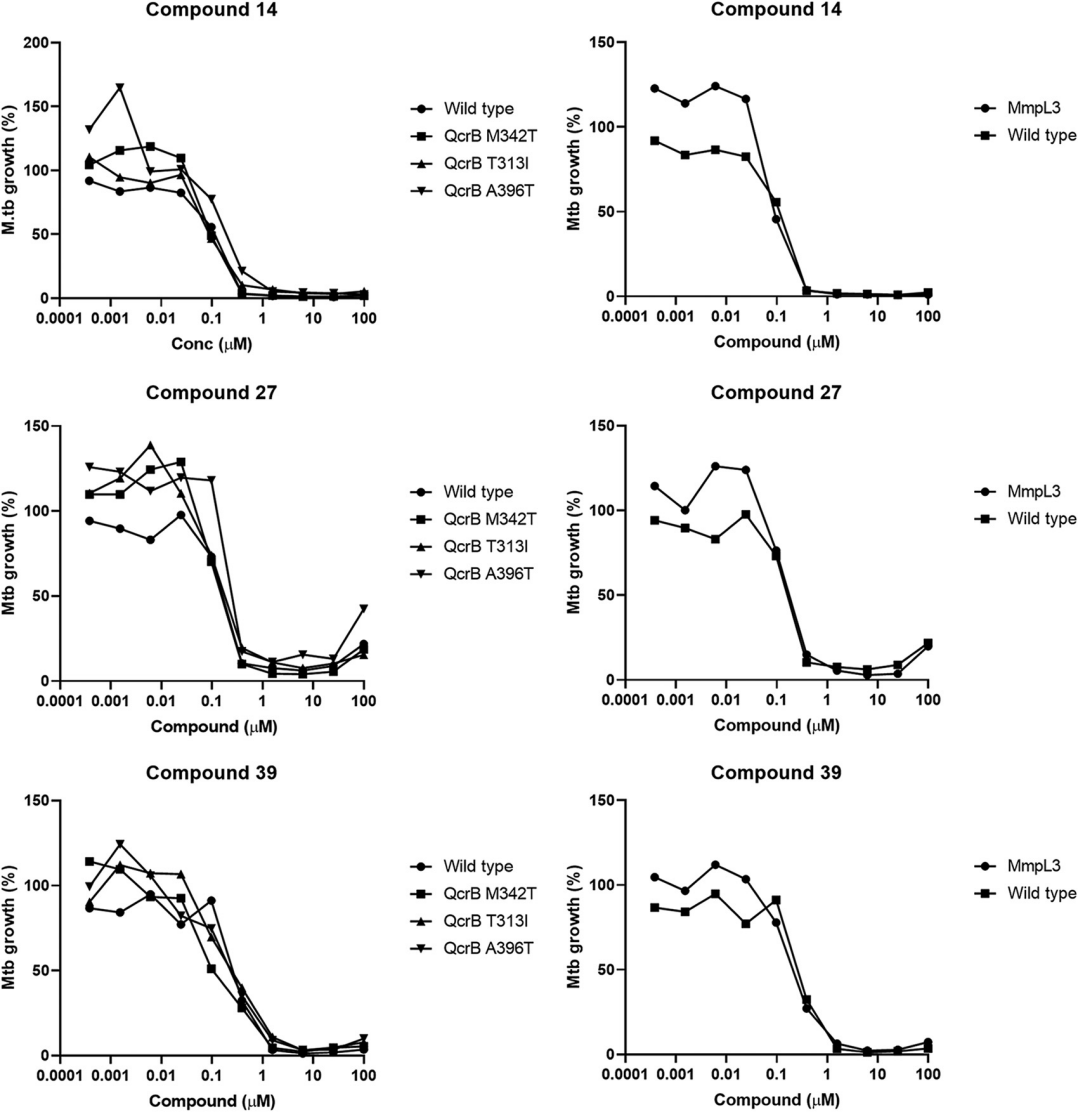
Activity against mutant strains of M. tuberculosis. Activity against intracellular M. tuberculosis strains carrying the indicated mutations was determined using the high-content assay. Mtb, M. tuberculosis.

Due to the promiscuous nature of several drug targets in M. tuberculosis (21), we tested three selected PTU compounds against a set of strains with mutations in the most promiscuous targets. We generated fluorescent versions (*Ds*Red) of well-characterized strains carrying mutations in either QcrB or MmpL3. For QcrB, we used strains with M342T, T313I, or A396T mutations (22); for MmpL3, we used strains with a triple mutation of F255L, V646M, and F644I (23). We determined IC_50_ values for a small set of compounds with good activity against these strains (Fig. 6). No shift in susceptibility was observed with any of the mutants tested, indicating that the PTU series does not exert its antibacterial activity through these established targets.

One of the best characterized, conditionally essential pathways for intracellular growth of M. tuberculosis is cholesterol metabolism (24, 25). The ability to utilize cholesterol is crucial for M. tuberculosis during macrophage infection (24–26). We determined whether the PTU compounds had increased activity in medium with cholesterol as the only carbon source. We tested three active compounds (Table 3). Two compounds showed no significant difference in activity from bacteria cultured with glucose (less than 2-fold change either way). One compound showed marginal increase in activity (3.9-fold more active), but this was slightly below our cutoff of 4-fold being a significant difference. These small changes indicate that inhibition of cholesterol metabolism is not a mechanism of action of this series.

**TABLE 3:** Biological activity against extracellular M. tuberculosis in cholesterol medium

Compound	Cholesterol^*a*^ IC_50_ (μM)	Glucose^*a*^ IC_50_ (μM)	Fold-change
14	15	7.2	0.5
16	6.6	26	3.9
22	5.7	11	1.9

a Activity against extracellular M. tuberculosis was determined in culture medium containing either cholesterol or glucose as a carbon source. IC_50_, concentration required to inhibit by 50%. Data are the average of at least two independent experiments.

## DISCUSSION

We explored the potential of the PTU series as antitubercular agents targeting intracellular bacteria. We tested 35 analogs which were chosen to determine key features required for activity and selectivity. We explored the role of the central thiourea and the extent to which the sides groups could be replaced, substituted, and removed. We found that the thiourea could be replaced with a urea and maintain activity but that this was detrimental to selectivity. We determined that the two phenyl groups are not both required for activity, but only replacements with aromatic groups were tolerated. Decoration of the phenyl rings with electron-withdrawing groups had a positive impact on activity with minimal effect on selectivity. Future exploration of this series would include plans to maintain the thiourea core, expand the number of aromatic groups, and focus efforts on optimizing properties with additional substitutions.

The PTU compounds exhibit activity against extracellular bacteria, although to a lesser extent than against intracellular bacteria. We do not know why compounds have differential activity against intracellular and extracellular activity, although there are several possibilities. Compounds could have additional effect on the host cell macrophage, or they could be metabolized or accumulate inside the host cell. Future work could address these possibilities. This dual activity makes them attractive and is likely related to the mechanism of action of this series.

Screens for inhibitors of intracellular replication have identified compounds either with increased activity or with selective activity. For example, another high-content screen identified inhibitors of DprE1, involved in cell wall biosynthesis (27). In this case, it was not clear why compounds were more active against intracellular bacteria, since DprE1 is also essential for extracellular growth. Compounds which target cholesterol metabolism are also differentially active in the macrophage. However, unlike our compounds, they have no activity against M. tuberculosis cultured with glucose as the sole carbon source (26). Host-activated molecules or host processes can also contribute to differential activity; for example, inhibitors of G-protein coupled receptor modulators, ion channels, membrane transporters, and kinases have been identified (28). Since the complement of essential genes is different between intracellular and extracellular bacteria (12–14), it is likely that vulnerability will also vary between conditions, and thus we may have hit a target which is more important for intracellular survival.

We identified this series of molecules using a high-content assay which was designed to select for compounds with improved intracellular activity and to minimize false positives resulting from macrophage cell death. While this assay measures bacterial growth in a robust fashion (signal:background > 5, signal:noise > 5, %CV (coefficient of variation) < 20, Z′ (Z prime) of controls > 0.5, minimum significant ratio > 3) and can be used to monitor inhibition of growth, it is not sensitive enough to monitor bacterial killing, which remains to be enumerated. In addition, where molecules are toxic to the macrophages, it becomes impossible to determine if they are active against the bacterial population (since they do not grow in the cell culture medium). Therefore, some molecules with activity could be missed or activity could be underestimated. One further limitation of the assay is its short-term nature, with only 3 days of exposure to compounds, such that molecules with delayed action could be missed. The main difficulty in developing longer-term infection models is to maintain the viability of infected macrophages over a longer time course.

Our initial mode of action investigation was designed to rule out common or well-established mechanisms. We were able to exclude common targets, which suggests that the PTU compounds exert antitubercular activity through a distinct or novel target and/or mechanism. Future work will focus on target identification and exploring the range of conditions under which this series is active.

## MATERIALS AND METHODS

### Culture.

Murine RAW 264.7 macrophages (ATCC TIB-71) were cultured in RPMI 1640 medium supplemented with 1 mM sodium pyruvate, 2 mM GlutaGro (Corning), and 5% fetal bovine serum. Human hepatic cells HepG2 (ATCC HB-8065) were cultured in Dulbecco’s modified Eagle’s medium (DMEM) with 10% fetal bovine serum and 1× penicillin streptomycin solution (100 U/mL). Cultures were maintained in a humidified incubator with 5% CO_2_ at 37°C. M. tuberculosis strains constitutively expressing codon-optimized DsRed from plasmid pBlazeC8 (DREAM8) (29) were cultured at 37°C in Middlebrook 7H9 medium containing 10% vol/vol OADC (oleic acid, dextrose, catalase) supplement (Becton, Dickinson) and 0.05% wt/vol Tween 80 (7H9-Tw-OADC) plus 100 μg/mL hygromycin B. M. tuberculosis H37Rv-LP (ATCC 25618) was the wild-type strain; strains containing mutations in QcrB (M342T, T313I, or A396T) (22) or MmpL3 (23) were derived from this parental strain as described previously. For growth on cholesterol, M. tuberculosis was cultured in Middlebrook 7H9 medium containing 0.1% cas-amino acids, 500 mg/L 2-morpholinoethanesulfonic acid, 5 g/L bovine serum albumin (BSA), 0.05% wt/vol tyloxapol, and 0.1 mM cholesterol (7H9-Tyl-Chol).

### Kill kinetics.

M. tuberculosis was inoculated into Middlebrook 7H9 medium containing 10% vol/vol/Middlebrook OADC supplement (Becton, Dickinson) and 0.05% wt/vol Tween 80 (7H9-Tw-OADC) at ~5 × 10^5^ CFU per mL. Compounds were prepared as 10 mM stocks in dimethyl sulfoxide (DMSO) and added to the indicated final concentration (final DMSO of 2%). Viable bacteria were determined over 21 days by preparing 10-fold serial dilutions (10^0^ to 10^−5^ dilution series) and plating onto Middlebrook 7H10 agar containing 10% vol/vol OADC. Plates were incubated for 21 to 28 days, and colonies were counted to determine CFU/mL (30).

### Determination of intracellular activity.

Compounds were purchased from standard commercial vendors. Activity against M. tuberculosis and RAW 264.7 cells was determined as described previously (11). Briefly, assay plates were prepared in clear-bottom, black, 384-well plates with 30 μL cRPMI and 0.6 μL compound (final concentration 1% DMSO); compounds were tested as 10-point serial dilutions to generate dose response curves. One percent DMSO was used as the negative control for maximum growth, and 10 mM isoniazid (INH) and 100 μM staurosporine (STA) were included as positive controls (maximum inhibition) for antitubercular activity and cytotoxicity, respectively. INH and STA were also included in dose response on each plate. RAW 264.7 cells were infected with M. tuberculosis DREAM8 at a multiplicity of infection (MOI) of 1 for 24 h, and extracellular bacteria was removed by washing. Cells were recovered using Accumax, harvested, washed, and resuspended in serum-free RPMI; 30 μL of infected cells was dispensed into each well at 3,300 cells/well. Plates were incubated for 72 h, 10 μL of 5× SYBR green I was added, and plates were imaged with an ImageXpress micro high-content screening system (Molecular Devices) using a 4× objective and fluorescein isothiocyanate (FITC) and Texas Red channels. MetaXpress was used to analyze images. Bacterial growth and macrophage survival were measured by fluorescence. Total fluorescence (integrated intensity) for each well was measured for both M. tuberculosis (red fluorescence) and macrophages (green fluorescence) using MetaExpress software (11). Growth inhibition for either M. tuberculosis or macrophages was calculated for each test well by normalizing to the average of the maximum growth wells (1% DMSO control). Curves were fitted using the Levenberg–Marquardt algorithm, and IC_50_ was calculated as the compound concentration required to reduce bacterial or eukaryotic cell growth by 50%.

### Cytotoxicity.

Cytotoxicity against HepG2 cells was measured after 72 h. Cells were seeded in 384-well plates at 1,800 cells per well. Compounds were added as a 10-point, 3-fold serial dilution after 24 h (final assay concentration of 1% DMSO). CellTiter-Glo reagent (Promega) was added, and relative luminescence units (RLU) were measured. Data were normalized to the DMSO controls. Curves were fitted using the Levenberg–Marquardt algorithm; IC_50_ was calculated as the compound concentration required to reduce HepG2 cell s by 50%.

### MIC.

Inhibitory concentrations (IC_50_) against M. tuberculosis were determined in liquid medium as described previously (31). Bacterial growth was measured after 5 days by optical density at 590 nm (OD_590_). IC_50_ was defined as the concentration of compound required to inhibit growth of M. tuberculosis by 50% and was determined using the Levenberg–Marquardt least-squares plot. For MICs in cholesterol, M. tuberculosis was precultured in 7H9-Tyl-Chol for 7 days and MICs were determined after 7 days of growth in 7H9-Tyl-Chol.
